# Phosphatidyl inositol-3 kinase (*PIK3CA*) E545K mutation confers cisplatin resistance and a migratory phenotype in cervical cancer cells

**DOI:** 10.18632/oncotarget.10955

**Published:** 2016-07-30

**Authors:** Wani Arjumand, Cole D. Merry, Chen Wang, Elias Saba, John B. McIntyre, Shujuan Fang, Elizabeth Kornaga, Prafull Ghatage, Corinne M. Doll, Susan P. Lees

**Affiliations:** ^1^ Department of Biochemistry and Molecular Biology, University of Calgary, Calgary, Alberta, Canada; ^2^ Robson DNA Science Centre, Arnie Charbonneau Cancer Institute, University of Calgary, Calgary, Alberta, Canada; ^3^ Translational Laboratory, Tom Baker Cancer Centre, Calgary, Alberta, Canada; ^4^ Department of Oncology, University of Calgary, Calgary, Alberta, Canada

**Keywords:** PI3K pathway, PIK3CA-E545K, Akt, GDC-0941, cisplatin

## Abstract

The phosphatidylinositol-3 kinase (PI3K)/Akt/mTOR signaling pathway is activated in many human cancers. Previously, we reported that patients with early stage cervical cancer whose tumours harbour *PIK3CA* exon 9 or 20 mutations have worse overall survival in response to treatment with radiation and cisplatin than patients with wild-type *PIK3CA*. The purpose of this study was to determine whether *PIK3CA-E545K* mutation renders cervical cancer cells more resistant to cisplatin and/or radiation, and whether PI3K inhibition reverses the phenotype. We found that CaSki cells that are heterozygous for the *PIK3CA*-E545K mutation are more resistant to cisplatin or cisplatin plus radiation than either HeLa or SiHa cells that express only wild-type *PIK3CA*. Similarly, HeLa cells engineered to stably express *PIK3CA*-E545K were more resistant to cisplatin or cisplatin plus radiation than cells expressing only wild-type *PIK3CA* or with *PIK3CA* depleted. Cells expressing the *PIK3CA*-E545K mutation also had constitutive PI3K pathway activation and increased cellular migration and each of these phenotypes was reversed by treatment with the PI3K inhibitor GDC-0941/Pictilisib. Our results suggests that cervical cancer patients whose tumours are positive for the *PIK3CA*-E545K mutation may benefit from PI3K inhibitor therapy in concert with standard cisplatin and radiation therapy.

## INTRODUCTION

Genetic analyses of tumours have shown that aberrant phosphatidyl inositol-3 kinase (PI3K) signaling is a critical oncogenic stimulus in many different types of cancer including breast, bladder, prostate, thyroid, ovarian and non-small-cell lung cancer (NSCLC) [1–6]. Class I PI3Ks are a family of lipid kinases that regulate signaling pathways involved in cell proliferation, transformation, cell survival, apoptosis and metastasis [7]. The Class I PI3K family is further divided into subclasses 1A and 1B where *PIK3CA*, *PIK3CB* and *PIK3CD* code for class 1A PI3Ks and *PIK3CG* codes for class 1B PI3Ks [8]. The product of the *PIK3CA* gene is a 110-kDa catalytic subunit (p110α) that exists in complex with a regulatory 85-kDa subunit (p85) [9]. In quiescent cells, p85 maintains the p110α catalytic subunit in a low-activity state. When active, or upon growth factor stimulation, the SH2 domain [Rous-sarcoma (src) oncogene homology-2 domain] of the p85 subunit binds to phosphorylated tyrosine in receptor tyrosine kinases, resulting in recruitment of p110α to the plasma membrane [10]. Active p110α phosphorylates phosphatidylinositol 4,5-bisphosphate (PIP2) to form phosphatidylinositol 3,4,5-triphosphate (PIP3), which recruits adaptor and effector proteins containing a pleckstrin homology domain (PH domain) to cellular membranes including the protein kinases Akt and phosphoinositide-dependent kinase-1 (PDK-1) [11].

Akt is a well-characterized serine/threonine kinase that promotes cellular survival and is activated in response to many different growth factors, including IGF2-I, epidermal growth factor, basic fibroblast growth factor, insulin, interleukin 3, interleukin 6, heregulin, and vascular endothelial growth factor [12]. Once at the membrane, Akt is phosphorylated at Thr308 and Ser473 by PDK1 and the mTORC2 complex, respectively [13]. Activated Akt phosphorylates and activates target proteins including GSK3β, BAD, MDM2, caspase 9, a subset of fork-head transcription factors and mTOR, which in turn regulate phosphorylation of p70-S6K, 4EBP1 and other target proteins to regulate cell survival, proliferation, cell cycle, protein synthesis and other cellular processes [14, 15].

The *PIK3CA* gene is amplified in many cancers including head and neck, lung, gastric and cervix [16]. In addition, many human cancers including cervical cancer, express p110α with activating mutations, with a reported prevalence ranging from 13-36% [17]. The majority of *PIK3CA* mutations cluster in “hotspots” in exon 9 (corresponding to the helical domain of PI3K p110α) and exon 20 (corresponding to the kinase domain of PI3K 110α). The most common missense mutations in *PIK3CA* result in replacement of glutamic acid 542 or 545 in the helical domain with lysine (E542K and E545K, respectively) or replacement of histidine 1047 in the kinase domain with arginine (H1047R) [18]. Activating mutations are associated with increased enzymatic activity independent of upstream signaling. Such constitutive activation stimulates signaling through the Akt pathway, and confers oncogenic properties such as increased cell invasion and metastasis [19]. *PIK3CA* mutations have also been associated with resistance to the microtubule poison paclitaxel as well as the Her2/neu antibody, trastuzumab/Herceptin [20, 21], suggesting that activation of the PI3K pathway may contribute to treatment resistance.

Based on the critical role of the PI3K/Akt/mTOR axis in the control of cell growth, metabolism and migration, components of this pathway represent attractive candidates for targeted cancer agents. Consequently, a number of potent and selective PI3K inhibitors have recently entered early-phase clinical trials [22]. Among them is GDC-0941/Pictilisib, a potent and selective inhibitor of class I PI3K with low nanomolar potency against all four class I isoforms. GDC-0941 binds to the ATP-binding pocket of PI3K p110, preventing formation of PIP3, activation of PI3K and phosphorylation of downstream targets such as Akt [23–25]. Moreover, GDC-0941 is under evaluation in phase I/II clinical trials in patients with advanced solid tumours [26] and is being evaluated in combination with cisplatin in patients with androgen receptor negative, triple negative breast cancer (https://clinicaltrials.gov/ct2/show/NCT01918306).

Previously we showed that a subset of cervical cancer patients with *PIK3CA* exon 9 or 20 mutations had significantly worse clinical outcomes after radiation therapy (RT) and cisplatin chemotherapy than patients whose tumours expressed wild-type *PIK3CA* [27]. In patients with *PIK3CA* mutations, approximately 60% were E545K positive [27]. Similarly, the *PIK3CA*-E545K mutation was reported to be highly prevalent in a large cohort of Latin American patients with cervical tumours [28]. The aim of the present study was to evaluate the effects of PI3K p110α-E545K mutation on Akt pathway activation and cell survival in cervical cancer cell lines treated with radiation and/or cisplatin, and to determine the effects of PI3K pathway inhibition on the cellular response to radiation and cisplatin treatment. Our results show that cervical cancer cells expressing *PIK3CA*-E545K are more resistant to cisplatin or cisplatin in combination with ionizing radiation (IR) than cells expressing *PIK3CA*-WT. Moreover, the PI3K inhibitor GDC-0941 inhibited phosphorylation of Akt and reversed the cisplatin-resistant phenotype in cervical cancer cells expressing *PIK3CA*-E545K. We also show that cervical cancer cells expressing *PIK3CA*-E545K have a more migratory phenotype than cervical cancer cells expressing *PIK3CA*-WT and this phenotype is also reversed by GDC-0941. Taken together, these observations suggest that not only is *PIK3CA* a potential drug target for the treatment of patients with cervical cancer, but its inhibition may improve the delivery and efficacy of standard cisplatin chemotherapy, reduce the migratory potential of tumour cells and ultimately improve patient outcomes in patients with *PIK3CA*-E545K mutation who receive cisplatin as part of their treatment regimen.

## RESULTS

### *PIK3CA*-E545K mutation confers resistance to cisplatin in cervical cancer cell lines

**Figure 1:**
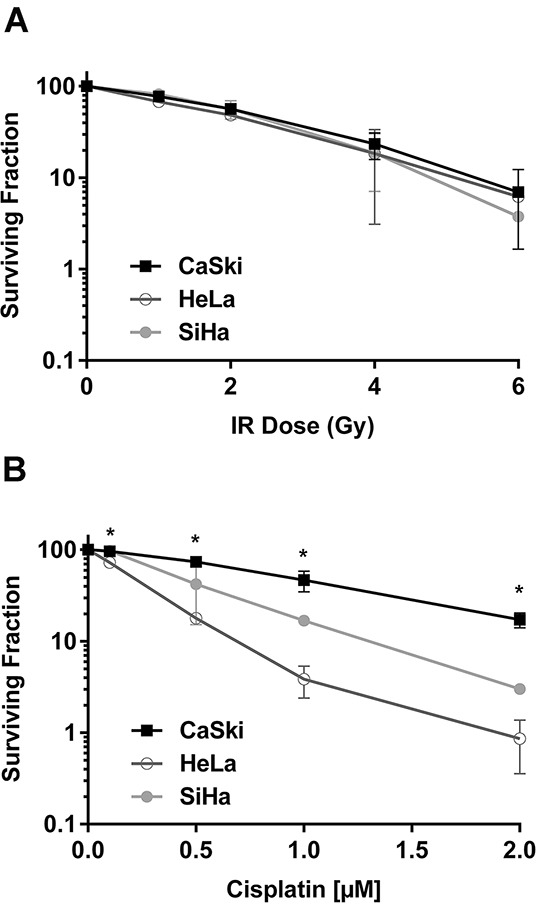
CaSki cells that express *PIK3CA*-WT and *PIK3CA*-E545K are resistant to cisplatin but not IR **A.** Cervical cancer cell lines HeLa (*PIK3CA-WT*) (open circles), SiHa (*PIK3CA*-WT) (closed circles) and CaSki (*PIK3CA*-WT plus *PIK3CA*-E545K) (squares), were seeded on 6 cm plates and 24 hours later irradiated at 1, 2, 4, and 6 Gy, respectively. Plates were incubated at 37°C under 5% CO_2_ for 14 days prior to staining using crystal violet, and surviving colonies (clusters of >50 cells) were counted. Each experiment was carried out in triplicate and means of 3 separate experiments ± S.E.M. (n=3) are plotted. No statistically significant differences were observed between the three cell lines at any IR dose. **B.** Cells were seeded on 6 cm plates and 24 hours later treated with control PBS or cisplatin formulated in PBS at 0.1, 0.5, 1 and 2 μM, respectively. Cisplatin was removed after 24 hours and replaced with fresh media. Plates were analysed after 14 days as described in Panel A. The *p* values for the 0.1, 0.5, 1 and 2 μM cisplatin data points for CaSki compared to HeLa were 0.0087, 0.0006, 0.0227 and 0.0071 respectively. *p* values < 0.05 were considered statistically significant and are indicated by asterisks.

To better understand the effects of *PIK3CA*-E545K mutation on cervical cancer cells, we first examined the effects of IR and cisplatin on survival of a panel of cervical cancer cell lines. For these studies, we selected HeLa (cervical adenocarcinoma derived cell line with *PIK3CA*-WT), SiHa (cervical squamous cell carcinoma cell line with *PIK3CA*-WT) and CaSki cells (a cervical squamous cell carcinoma cell line that is heterozygous for *PIK3CA*-E545K, Supplementary Table S1). Importantly, in all experiments cisplatin was made up and stored in PBS, consistent with a recent report that cisplatin is inactivated when reconstituted in DMSO [29]. No significant differences were observed in survival of HeLa, SiHa or CaSki cells after exposure to 1-6 Gy IR (Figure 1A), however CaSki cells were significantly more resistant to cisplatin than either HeLa or SiHa cells (Figure 1B). Specifically, CaSki cells showed ~30% survival at 2 μM cisplatin, compared to ~3% and ~1% in SiHa and HeLa respectively, suggesting that expression of *PIK3CA*-E545K may confer resistance to cisplatin in cervical cancer cells.

**Figure 2:**
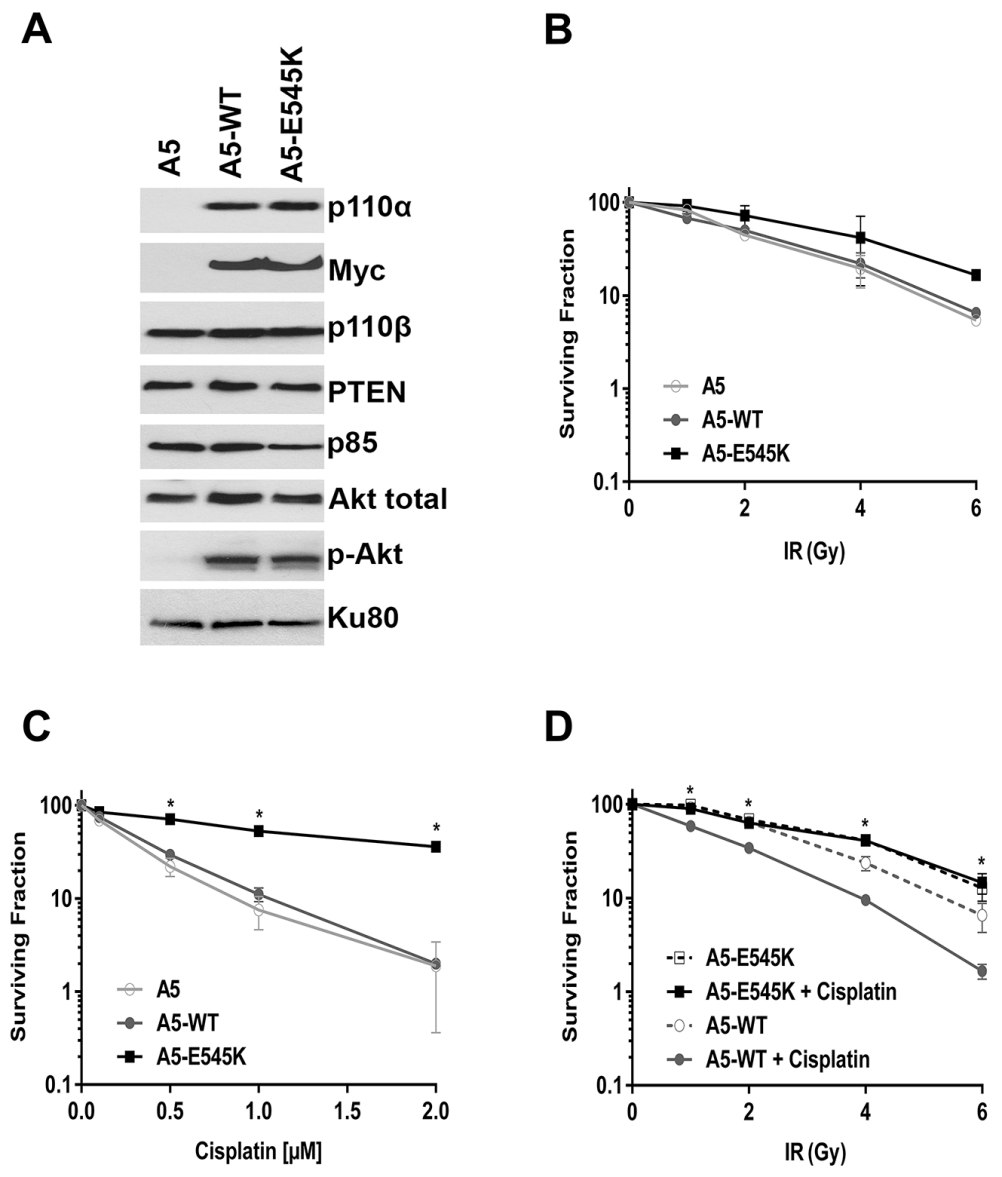
HeLa cells expressing *PIK3CA*-E545K are resistant to cisplatin **A.** HeLa-A5 cells (shRNA depletion of *PIK3CA*), A5 cells transfected with shRNA resistant *PIK3CA*-WT (A5-WT), and A5 cells transfected with shRNA-resistant *PIK3CA*-E545K (A5-E545K) were grown under standard conditions (5% serum). Whole cell extracts were generated and aliquots containing 50 μg protein were run on SDS PAGE. Western blots were probed with antibodies to p110α, p110β, p85, Akt, Akt-pS473, PTEN, Myc (to detect stably incorporated Myc-tagged *PIK3CA*) and Ku80 (loading control). Quantitation of protein levels relative to the loading control (Ku80) is shown in Supplementary Figure 2A. **B.** A5 (shRNA depletion of *PIK3CA* in HeLa cells) (open circles), A5 cells transfected with shRNA resistant *PIK3CA* WT type (A5-WT) (closed circles), and A5 cells transfected with shRNA-resistant *PIK3CA*-E545K (A5-E545K) (closed squares) were either unirradiated (0), or treated with different doses of IR (1, 2, 4 and 6 Gy). Clonogenic survival assays were carried out as described in Figure 1. No statistically significant differences were observed between the three cell lines at any dose tested. **C.** A5, A5-WT, and A5-E545K cells as in panel B were seeded on 6 cm plates and 24 hours later treated with either control PBS (containing 154 mM NaCl) or cisplatin (formulated in PBS) at 0.1, 0.5, 1 or 2 μM, respectively. Cisplatin was removed after 24 hours and replaced with fresh media. Clonogenic survival assays were carried out as above. At 0.1, 0.5, 1.0 and 2 μM cisplatin, the *p* values for A5-WT compared to A5-E545K were 0.0288, 0.0004, 0.0016 and <0.0001, respectively. *p* values of <0.05 were considered statistically significant and are indicated by the asterisks. **D.** A5-WT cells (open and closed circles) and A5-E545K cells (open and closed squares) were seeded on 6 cm plates and 24 hours later either mock treated with PBS (open symbols and dashed lines) or treated with cisplatin (formulated in PBS) at 1 μM (closed symbols, solid lines). Cisplatin was removed after 24 hours and replaced with fresh media, then cells were either unirradiated (0) or irradiated at 1, 2, 4, and 6 Gy, respectively. After 14 days, the plates were fixed, stained and colonies were counted and analyzed as above. At 1, 2, 4 and 6 Gy plus cisplatin data points, the p values for A5-WT + cisplatin compared to A5-E545K + cisplatin were <0.0001, <0.0001, 0.00045, and 0.023, respectively. *p* values of <0.05 were considered statistically significant and are indicated by the asterisks.

Because cancer cell lines likely bear multiple mutations that could confound interpretation of results, it was important to determine the effects of *PIK3CA*-E545K mutation in an otherwise isogenic background. We therefore depleted endogenous *PIK3CA* from HeLa cells using shRNA (to generate A5 cells) then reintroduced either shRNA-resistant *PIK3CA-*WT (A5-WT cells) or shRNA-resistant *PIK3CA*-E545K (A5-E545K cells. Supplementary Table S1 and Supplementary Figure S1). Whole cell lysates were generated from asynchronously growing A5, A5-WT and A5-E545K cells and aliquots were analyzed by western blot for the presence of the catalytic and regulatory subunits of PI3K and phosphorylation of Akt. The results confirm that A5 cells have no detectable p110α expression while p110α was expressed at similar levels in A5-WT and A5-E545K cells (Figure 2A and Supplementary Figure S2A). Expression of p110β, p85 and PTEN was also similar between the three cell lines tested indicating that loss of *PIK3CA* expression (A5 cells) does not affect expression of other components of the pathway (Figure 2A and Supplementary Figure S2A). Total Akt protein was present in all cell lines generated, however no phosphorylation of Akt-S473 was observed in A5 cells, consistent with lack of *PIK3CA* expression. Myc expression confirmed expression of plasmids for *PIK3CA*-WT and E545K (Figure 2A).

To determine whether expression of *PIK3CA*-E545K in an isogenic background conferred resistance to IR and/or cisplatin, A5, A5-WT and A5-E545K cells were exposed to either different doses of IR (1, 2, 4 and 6 Gy) (Figure 2B) or different doses of cisplatin (0.1, 0.5, 1 and 2 μM, reconstituted in PBS, Figure 2C) and survival was determined using clonogenic survival assays. Our results show that A5-E545K cells are more resistant to cisplatin than either A5-WT or A5 cells (Figure 2C), whereas cellular survival after IR was similar in the three cell lines tested (Figure 2B).

Since the standard of care for cervical cancer patients is cisplatin in combination with RT, we also examined the effects of IR combined with cisplatin on cell survival. Cells were incubated with cisplatin (1 μM in PBS) or an equivalent volume of PBS for 24 hours then cisplatin was removed and replaced with fresh media. Cells were then either unirradiated (0 Gy), or irradiated with 1-6 Gy and 14 days later, survival determined using clonogenic survival assays as above. We found that A5-WT cells were sensitive to cisplatin treatment in the presence of IR, whereas A5-E545K cells were resistant to cisplatin, even when co-treated with IR (Figure 2D). Together, these data strongly suggest that expression of *PIK3CA*-E545K in cervical cancer cells confers resistance to cisplatin and cisplatin plus IR treatment but not IR alone.

**Figure 3:**
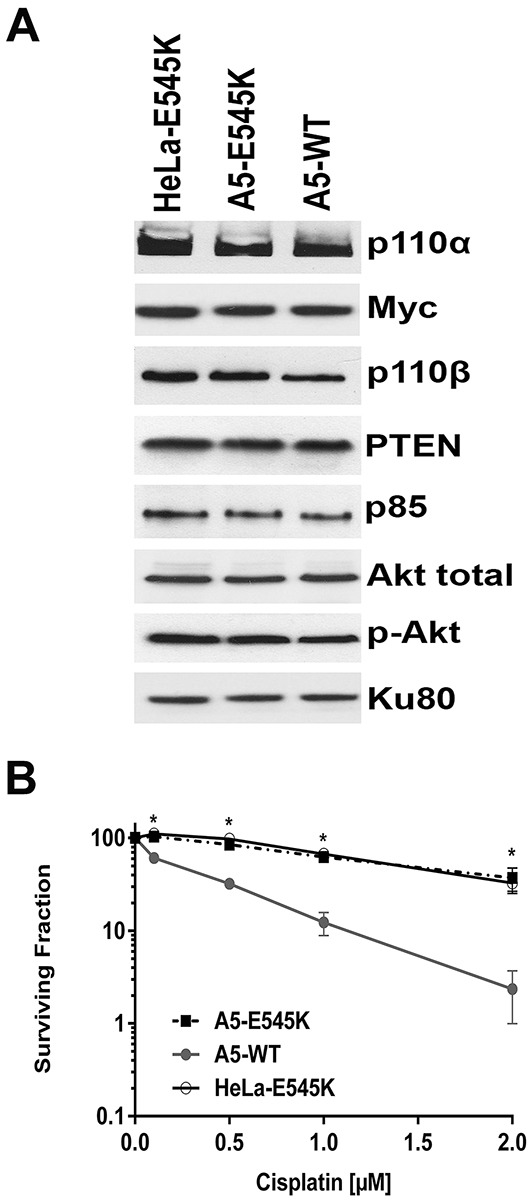
HeLa cells stably expressing both *PIK3CA*-E545K and *PIK3CA*-WT are resistant to cisplatin **A.** HeLa cells stably expressing Myc/DDK-tagged *PIK3CA*-E545K (HeLa-E545K) were generated as described in Materials and Methods. Aliquots containing 50 μg protein were run on SDS PAGE, immunoblotted and probed with antibodies to p110α and β, Myc (to detect transfected *PIK3CA*-E545K), p85, PTEN and Ku80 (loading control). Quantitation of protein levels relative to the loading control (Ku80) is shown in Supplementary Figure S2B. **B.** A5-WT (closed circles), A5-E545K (closed squares) and HeLa cells overexpressing *PIK3CA*-E545K (HeLa-E545K) (open circles) were seeded on 6 cm plates and 24 hours later treated either with PBS (154 mM NaCl) as control, or cisplatin (formulated in PBS) at 0.1, 0.5, 1 or 2 μM. Cisplatin was removed after 24 hours and replaced with fresh media and plates were analysed by clonogenic survival assays as in Figure 2. For A5-WT and A5-E545K, at the 0.1, 0.5, 1 and 2 μM cisplatin data points, the *p* values were 0.010, 0.010, 0.0034 and 0.0301 respectively. Similarly, for HeLa-E545K and A5-WT, the *p* values for 0.1, 0.5, 1 and 2 μM cisplatin data points were 0.0052, 0.0024, 0.0038 and 0.017 respectively. *p* values of <0.05 was considered statistically significant and are indicated by the asterisks.

**Figure 4:**
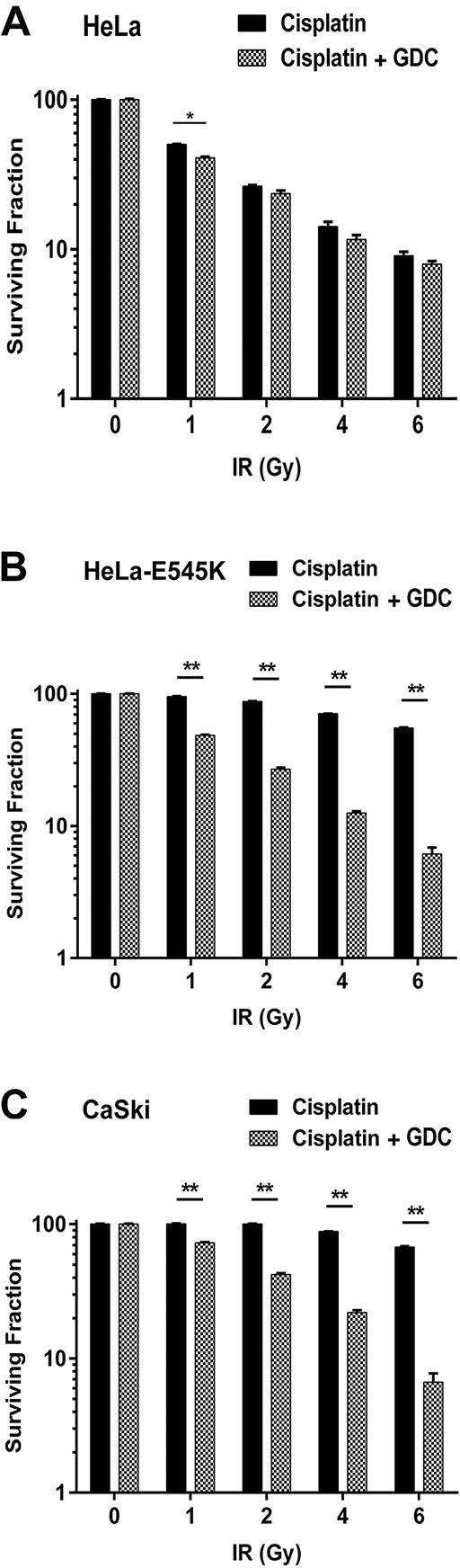
GDC-0941 restores cisplatin sensitivity in *PIK3CA*-E545K expressing cells Cervical cancer cell lines HeLa (*PIK3CA-WT,* panel **A.** HeLa cells overexpressing *PIK3CA*-E545K (HeLa-E545K, panel **B.** and CaSki (*PIK3CA*-WT plus *PIK3CA*-E545K, panel **C.** were seeded on 6 cm plates and 24 hours later treated with cisplatin (formulated in PBS) at 1 μM and GDC-0941 (formulated in DMSO) at 0.5 μM (final concentration 0.01% DMSO) or 0.01% DMSO alone, as indicated by the dark and gray/hatched bars, respectively. After 24 hours, media was replaced with fresh media and then the cells were either unirradiated (0) or irradiated at 1, 2, 4, and 6 Gy, as indicated. Plates were incubated at 37°C, under 5% CO_2_ for 14 days, then fixed, stained and colonies were counted as above. Statistical significance was determined using multiple t-tests between cisplatin plus IR and cisplatin plus GDC-0941 at each dose of IR. For HeLa cells, p values were 0.00037, 0.082, 0.12 and 0.19 respectively. For HeLa-E545K and CaSki cells, all values were <0.0001 and are represented by **.

Sequencing of the ~60% of patient tumour cells with *PIK3CA*-E545K mutation revealed that in at least 80% of the cases, the mutation was heterozygous i.e. patient tumour cells expressed both *PIK3CA-WT* and *PIK3CA*-E545K [27]. To more closely model the patient mutations, we stably expressed *PIK3CA*-E545K in parental HeLa cells that express endogenous *PIK3CA-WT*. Stable cells lines expressing both *PIK3CA-WT* and *PIK3CA*-E545K were isolated (HeLa-E545K, Figure 3A, Supplementary Figure S1C and Supplementary Figure S2B) and clonogenic survival assays were performed as above. Consistent with results in CaSki (Figure 1B) and A5-E545K cells (Figure 2C), HeLa-E545K cells were also resistant to cisplatin (Figure 3B), indicating that heterozygosity of *PIK3CA*-E545K in an otherwise isogenic cell line is sufficient for cisplatin resistance. We next compared sensitivity to cisplatin when combined with IR in control HeLa cells (*PIK3CA*-wt, Figure 4A), HeLa-E545K (stable expression of *PIK3CA*-E545K in HeLa cells, Figure 4B) and CaSki (heterozygous for *PIK3CA-E545K*, Figure 4C). As seen for cervical cancer cells homozygous for the *PIK3CA*-E545K mutation (Figure 2D), cervical cancer cells expressing *PIK3CA*-E545K in combination with *PIK3CA*-wt (HeLa-E545K and CaSki) were more resistant to cisplatin in combination with IR than HeLa cells expressing only *PIK3CA*-WT (Figure 4 compare black bars in panels A to black bars in panels B and C), confirming that heterozygous expression of *PIK3CA*-E545K confers resistance to cisplatin, even in the presence of IR.

### Expression of *PIK3CA*-E545K results in constitutive activation of the AKT pathway in cervical cancer cell lines

Previous studies in NSCLC and breast cancer cells have shown that the *PIK3CA*-E545K mutation results in constitutive activation of the PI3K pathway and enhanced signaling through the Akt pathway [30, 31]. To determine whether cervical cancer cells bearing the *PIK3CA*-E545K mutation also displayed enhanced PI3K signaling, A5-WT, A5-E545K and HeLa-E545K cells were grown under reduced serum conditions and AKT phosphorylation was assessed by immunoblotting with a phosphospecific antibody against the mTORC2 phosphorylation site Akt-S473. Phosphorylation of RSK at S380 was also evaluated as an indication of down regulation of ERK signaling in response to serum starvation [32]. In A5 cells (HeLa expressing *PIK3CA-WT)* grown in 0.2% serum (serum starved conditions), phosphorylation of Akt-S473 and RSK-S380 was reduced by at least 80% compared to the same cells grown under 5% serum (A5-WT, Supplementary Figure S3, lanes 1 and 2), whereas serum-starved A5-E545K cells (Supplementary Figure S3A and S3C, lanes 3 and 4) or HeLa-E545K cells (Supplementary Figure S3B and S3D, lanes 3 and 4) showed higher levels of both Akt-S473 phosphorylation (50-60%) and RSK-S380 phosphorylation (60-80%) after serum starvation, consistent with constitutive activation of the PI3K/Akt pathway in these cells. Our results support previous studies [13] showing that *PIK3CA*-E545K mutation results in constitutive activation of the Akt pathway in human cells. Since activating mutations in *PIK3CA* have been associated with resistance to various chemotherapeutic agents [20, 21], these results suggested a potential mechanism of cisplatin resistance in cervical cancer cells expressing *PIK3CA*-E545K and support the hypothesis that inhibiting the PI3K pathway may restore cisplatin sensitivity in cervical cancer cells expressing *PIK3CA*-E545K.

### Inhibition of the PI3K/Akt pathway with GDC-0941 sensitizes *PIK3CA*-E545K expressing cells to cisplatin

GDC-0941 is a class I selective PI3K inhibitor that has been shown to suppress PI3K and Akt signaling in breast cancer cell lines with PI3K pathway activation [33]. We therefore wanted to determine whether inhibition of PI3K/Akt signaling by GDC-0941 in cervical cancer cells expressing *PIK3CA*-E545K restored sensitivity to cisplatin and/or cisplatin + IR. To determine the optimal dose of GDC-0941 to use in our experiments, we first incubated cells with various concentrations of GDC-0941 for 6-72 hours, harvested cells and probed for Akt-pS473 phosphorylation. Since GDC-0941 is freely soluble in DMSO but not in water or ethanol (http://www.selleckchem.com/products/GDC-0941.html), for all experiments, GDC-0941 was made up as a stock solution in DMSO and diluted in cell media to give a final concentration of 0.5, 1 or 2 μM and, in each case, a final DMSO concentration of 0.01%. Control reactions contained 0.01% DMSO alone. Incubation with 0.5, 1 or 2 μM GDC-0941 reduced Akt-pS473 phosphorylation indicating it inhibits the PI3K/Akt pathway in our panel of cervical cancer cells (Supplementary Figure S4).

To determine the effect of GDC-0941 on survival in the presence of cisplatin or cisplatin plus IR, cells were incubated with cisplatin (made up in PBS) in the presence of either 0.5 μM GDC-0941 (made up in DMSO to give a final concentration of 0.01%) or an equivalent volume of DMSO (corresponding to a final concentration of 0.01%). Importantly, cisplatin has been shown to retain activity when incubated in media in the presence of 0.01% DMSO [29]. After 24 hours, media was removed, replaced with fresh media and cells were either unirradiated (0 Gy) or irradiated with 1-6 Gy and percent cell survival determined using clonogenic survival assays. As shown in Supplementary Figure S5, incubation of cells with 0.5 μM GDC-0941 alone for 24 hours reduced survival by approximately 40% in both CaSki and HeLa-E545K while only reducing survival by about 10% in HeLa cells, suggesting that GDC-0941 is more toxic to cervical cancer cell lines with the *PIK3CA*-E545K mutation, and consequent misregulated Akt signaling, than cells with endogenous *PIK3CA* signaling.

Significantly, GDC-0941 restored sensitivity in both HeLa-E545K (hatched bars, Figure 4B) and CaSki cells (hatched bars, Figure 4C) that had been treated with cisplatin plus IR, with little effect on control HeLa cells (Figure 4A). Similar results were seen in A5-WT and A5-E545K cells treated with cisplatin and GDC-0941 (Supplementary Figure S6). Together, these data show that cervical cancer cells bearing the *PIK3CA*-E545K mutation have activated PI3K signaling and resistance to cisplatin that can be overcome by inhibition of PI3K with GDC-0941.

### *PIK3CA*-E545K cells have an enhanced migratory phenotype, which can be suppressed by treatment with GDC-0941

**Figure 5:**
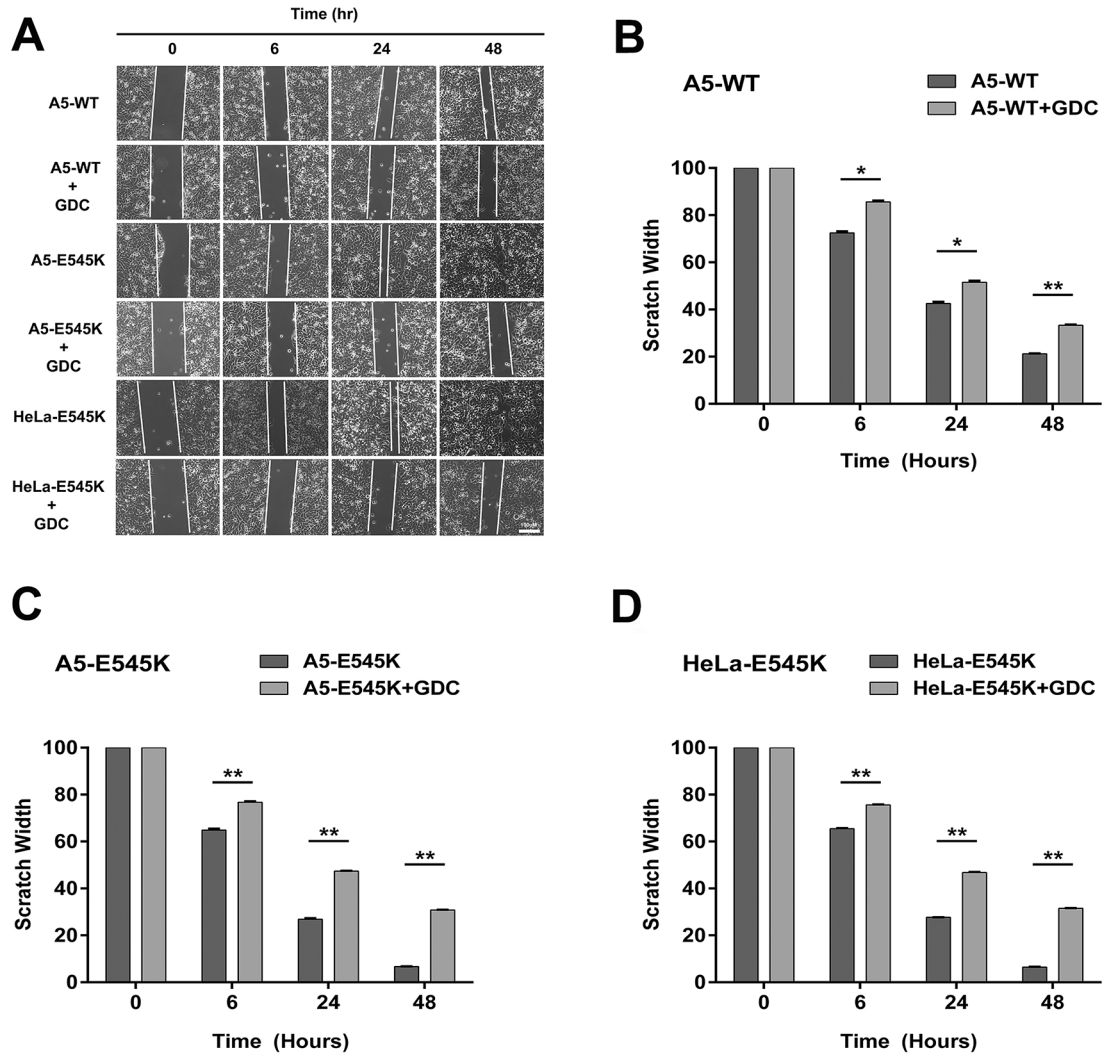
Stable cells expressing *PIK3CA*-E545K have a more migratory phenotype that is reversed by GDC-0941 **A.** A5-WT, A5-E545K and HeLa-E545K were grown to confluency on 6 cm plates and pre-treated with 1 μM GDC-0941 (formulated in DMSO) or an equivalent volume of DMSO (final concentration 0.01%) for 24 hours and the next day scratches were made and wound healing was observed as described in Materials and Methods. Representative images are shown. Scale bar = 100 μm. **B.** Average scratch widths, normalized to the width at 0 hours, from three separate experiments with standard deviation are shown using multiple comparison tests. For each cell line the reduction in the width of the scratch ± GDC-0941 at 6, 24 and 48 hrs was compared. For A5-WT p values were 0.00011, 0.00065 and <000.1 respectively. P values < 0.005 are indicated by ** and < 0.0001 by ***. **C.** As in panel B but for A5-E545K cells minus and plus GDC-0951. **D.** As in panel B but for HeLa-E545K cells minus and plus GDC-0941 at 6, 24 and 48 hrs.

**Figure 6:**
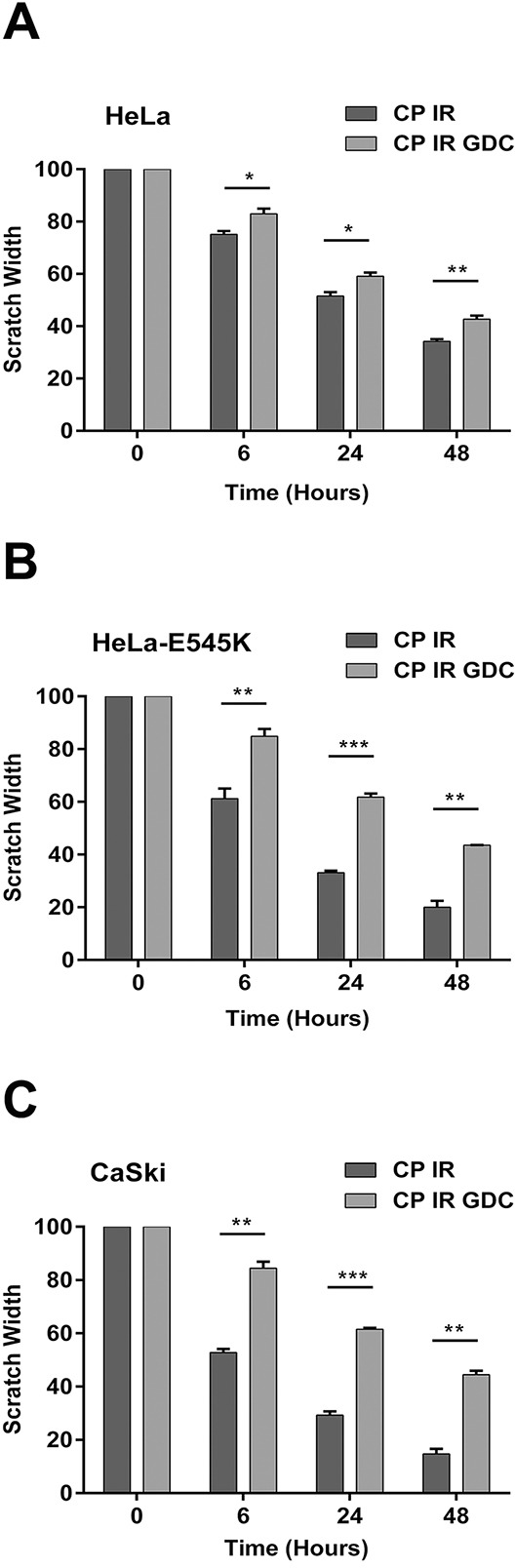
Cervical cancer cells expressing *PIK3CA*-E545K have a more migratory phenotype in scratch assays after cisplatin/IR treatment that is reversed by GDC-0941 Scratch assays were carried out as in Figure 5, but cells were either untreated or pre-treated with 1 μM GDC-0941 (formulated in DMSO) or an equivalent volume of DMSO (final concentration 0.01%), radiation (2Gy) and cisplatin (formulated in PBS) at 1 μM for 24 hours. Scratches were made and wound healing was observed 0, 6, 24, and 48 hours after initiation of the scratch. For HeLa cells **panel A.** treated with cisplatin plus IR compared to cisplatin plus IR plus GDC-0941 at 6, 24, and 48 hours, the p values were 0.031, 0.02, 0.0057, respectively. For HeLa-E545K cells **panel B.** treated with cisplatin plus IR compared to cisplatin plus IR plus GDC-0941 at 6, 24, and 48 hours, the p values were 0.0076, <0.0001, and 0.00068, respectively. C. For CaSki cells **panel C.** treated with cisplatin plus IR compared to cisplatin plus IR plus GDC-0941, at 6, 24, and 48 hours, the p values were 0.00032, <0.0001, and 0.00027, respectively.

Previous studies have shown that activating mutations in *PIK3CA* promote invasion of human cancer cells and that cells expressing *PIK3CA*-E545K migrate faster in cell-based assays [14]. To determine the effects of *PIK3CA*-E545K mutation on cell migration in our cervical cancer cell lines, scratch assays were performed on confluent cells (as described in Materials and Methods) and cells were allowed to recover for variable lengths of time, up to 48 hours. Our results suggest that *PIK3CA*-E545K expressing cells migrate across the scratch faster than cells expressing *PIK3CA*-WT (Supplementary Figure S7), and that cell migration is attenuated in the presence of GDC-0941, particularly in cells expressing *PIK3CA*-E545K (Figure 5). Moreover, GDC-0941 reduced cell migration in cells treated with cisplatin and IR. The relative scratch width was greater in HeLa-E545K and CaSki cells than HeLa cells at all time points, consistent with GDC-0941 inhibition of both constitutively active PI3K and endogenous PI3K (Figure 6).

We concluded that the differences in migration pattern were unlikely to be due to changes in proliferation since the cell lines had similar growth curves by trypan blue staining when grown under normal conditions with 5% serum (Supplementary Figure S8). We also considered it possible that the cells failed to migrate across the wound because of drug or IR-induced cell death. To test for this possibility, experiments were set up as for scratch assays, but percent viability was determined using trypan blue exclusion assays. As shown in Supplementary Figure S9, 48 hours post scratch, viability in Hela cells treated with cisplatin/IR and GDC-0941 was approximately 90% compared to cells treated with cisplatin and IR without GDC. In contrast, in CaSki and HeLa-E545K, the % viability was reduced to about 70% after GDC treatment. Thus, we cannot exclude the possibility that part of the reason for decreased migration across the scratch in GDC-0941 treated cells was in part due to cell death. Nevertheless, these experiments confirm our findings that cells expressing *PIK3CA*-E545K are more sensitive to GDC-0941 than those expressing *PIK3CA*-WT.

**Figure 7:**
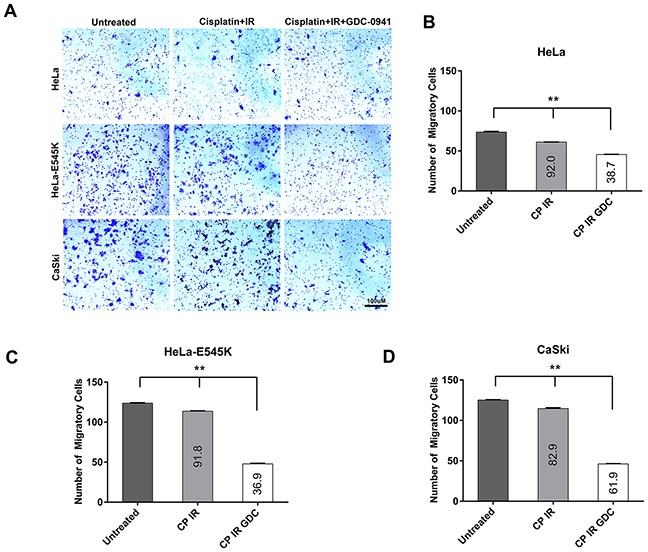
Cervical cancer cells expressing *PIK3CA*-E545K have a more migratory phenotype in transwell migration assays **A.** The migratory properties of cervical cancer cell lines HeLa (*PIK3CA-WT*), HeLa cells overexpressing *PIK3CA*-E545K (HeLa-E545K) and CaSki (*PIK3CA*-WT plus *PIK3CA*-E545K) were analyzed by a migration assay using a Transwell Boyden chamber system. Cells were either untreated or pre-treated with 1 μM GDC-0941 (formulated in DMSO) or an equivalent volume of DMSO (final concentration 0.01%), radiation (2Gy) and cisplatin (formulated in PBS) at 1 μM for 24 hours. Next day cells were seeded in the top chamber of the transwell migration apparatus. Media supplemented with 10% FBS was used as a chemoattractant in the lower chamber. Cells were incubated for 24hr at 37°C then non-migrated cells were removed from the upper face of the transwell insert using a cotton swab. Cells that migrated through membranes were stained with 1% crystal violet, 95% ethanol and then counted. Data are presented as means ± S.E.M. from three different experiments. Scale bar = 100 μm. **B.** Quantitation of results from transwell migration assays in HeLa (*PIK3CA-WT*). Different treatment conditions were compared using ordinary one-way ANOVA and Tukey's multiple comparisons test. *p* values were <0.0001 for untreated vs cisplatin + IR group; untreated vs cisplatin + IR + GDC-0941; and cisplatin + IR + GDC-0941, indicated by **. The percent of migratory cells as a % of untreated cells is shown on the graph. **C.** Quantitation of results from transwell migration assays in HeLa cells overexpressing *PIK3CA*-E545K (HeLa-E545K). Statistical comparisons were as in panel A. **D.** Quantitation of results from transwell migration assays in CaSki (*PIK3CA*-WT plus *PIK3CA*-E545K) cells, as in panels A and B.

Finally, to confirm results from scratch assays, we performed transwell migration assays (as described in Materials and Methods). Consistent with results from scratch assays, cervical cancer cells carrying the *PIK3CA*-E545K mutation (HeLa-E545K, Figure 7C, and CaSki, Figure 7D), displayed enhanced migration compared to cells expressing wild type *PIK3CA*. Moreover, as in scratch assays, treatment with GDC-0941 (in combination with radiation and cisplatin) reduced cell migration in CaSki and HeLa-E545K cells (Figure 7). Together, these results reveal that expression of *PIK3CA*-E545K in cervical cancer cell lines promotes both cisplatin resistance and a cell migratory phenotype and that both cisplatin resistance and cell migration can be mitigated at least in part, by inhibition of the PI3K pathway using GDC-0941.

## DISCUSSION

Despite the introduction of vaccines to high-risk human papilloma viral strains, cervical cancer remains a major source of cancer death, particularly in under-developed countries. Indeed, it is the third most common cancer diagnosis in women worldwide, and the fourth leading cause of cancer deaths [34]. The standard-of-care for locally advanced cervical cancer patients is radical radiation therapy (RT) in combination with cisplatin chemotherapy, yet 30-40% of patients are not cured of their disease. Cisplatin is also the most common chemotherapeutic agent used with RT in the adjuvant post-surgical setting for patients with cervical cancer. Although the addition of cisplatin chemotherapy to RT has been shown to improve survival vs RT alone, this comes at the expense of increased toxicity [35, 36]. Moreover, the mechanisms of chemoradiotherapy resistance remain poorly understood.

We have previously shown that patients with early stage cervical cancer (IB/II) with *PIK3CA* exon 9 or 20 mutations treated with radical RT in combination with cisplatin had significantly worse outcome than those whose tumours expressed *PIK3CA-WT* [27]. The majority of these mutations (almost 60%) were *PIK3CA*-E545K, and in approximately 80% of cases the mutation was heterozygous. A similar frequency of E545K mutation was reported recently in a large cohort of Latin American patients with cervical tumours [28]. Here we show that the CaSki cervical cancer cell line that is heterozygous for *PIK3CA*-E545K, is significantly more resistant to cisplatin than SiHa and HeLa that express *PIK3CA*-WT. Similarly, in HeLa cells in which the endogenous *PIK3CA* transcript was knocked down using shRNA and then restored with stably expressed shRNA-resistant *PIK3CA-*WT or shRNA-resistant *PIK3CA*-E545K, as well as HeLa cells expressing both *PIK3CA-*WT and *PIK3CA*-E545K, expression of *PIK3CA*-E545K correlated with cisplatin resistance and a more migratory phenotype, even when cells were co-treated with IR. Moreover, the selective class I PI3K inhibitor, GDC-0941 restored cisplatin sensitivity and slowed cellular migration in *PIK3CA*-E545K expressing cells. *PIK3CA*-E545K expression appeared to have a dominant phenotype since cisplatin resistance and enhanced migration were observed in cells expressing *PIK3CA*-E545K plus *PIK3CA*-WT as well as *PIK3CA*-E545K alone. Reduced cellular viability and migration was also observed in *PIK3CA*-WT expressing cells treated with GDC-0941, suggesting that GDC-0941 down-regulates signaling from endogenous PI3K signaling pathways, not only when it is over activated by E545K mutation.

Taken together, our studies suggest that constitutive activation of PI3K through E545K mutation leads to activation of Akt, likely through the mTORC2 pathway, as evidenced by enhanced phosphorylation of Akt-pS473 [15]. Activation of the PI3K pathway has been linked to the resistance of multiple chemotherapeutic drugs. The mechanism is poorly understood, but has been proposed to involve Akt-dependent activation of pro-survival pathways [37]. Cisplatin is a widely used chemotherapeutic agent that acts as a crosslinking agent, producing bulky adducts that can be repaired by nucleotide excision repair [38], Fanconi anemia and homologous recombination DNA repair pathways [39]. Mechanisms of resistance frequently involve altered DNA repair pathways or mechanisms of cisplatin uptake/excretion as well as up regulation of cellular pathways to inactivate cisplatin [38]. Precisely how these pathways might be affected by PI3K activation in cervical cancer cells will be an important area for further investigation.

Cell migration is a complex and highly coordinated process that is important for diverse biological processes, including embryonic morphogenesis, immune surveillance, tissue homoeostasis and wound healing [40], with PI3K signaling known to play a role in cellular migration [41]. Our results suggest that the *PIK3CA*-E545K mutation is associated with an enhanced migratory phenotype in cervical cancer cells. These results are consistent with earlier studies in which normal human urothelial cells expressing *PIK3CA*-E545K were shown to have accelerated migration, compared to cells with wild type *PIK3CA* [42]. Moreover, in the breast cancer cell line MDA-MB-231 modified to express either *PIK3CA*-E545K or H1047R, both displayed increased motility, with cells expressing *PIK3CA*-E545K showing increased directional migration [43]. Furthermore, functional analyses have shown that Akt can regulate migration and invasive processes [44–46].

The frequent activation of the PI3K pathway in many human cancers has led to intensive efforts to identify therapeutic agents that abrogate PI3K signaling and hence these agents may have clinical utility in patients with cancers that have an activated PI3K pathway. Numerous potent and selective PI3K inhibitors have recently entered early-phase clinical trials. Among them is GDC-0941, a PI3K pathway-inhibiting drug that is in advanced stages of clinical development. GDC-0941 is an oral class I pan-PI3K inhibitor with activity in the nanomolar range against a wide range of cancer cell lines that has preclinical activity in models harbouring pathway alterations in *PIK3CA*, *PTEN*, or *HER2* [24]. Currently GDC-0941 is in a phase II clinical trial in breast cancer patients [47]. Our studies showed that both enhanced migration and cisplatin resistance were abrogated by incubation of cervical cancer cell lines with GDC-0941. We also found that levels of Akt-pS473 were decreased by GDC-0941 treatment in a dose-dependent manner. Similarly, GDC-0941 has been shown to reduce RSK p380 phosphorylation in breast cancer cells expressing p110β activating mutations [48]. Our results also show that GDC-0941 was more toxic in cells expressing *PIK3CA*-E545K than cells expressing *PIK3CA*-WT, perhaps reflecting oncogene addiction in E545K expressing cells. Many PI3K pathway inhibitors as well as selective inhibitors of other components of the PI3K/AKT pathway are currently available, many in clinical trials [49]. It will be interesting to determine the effects of other PI3K/AKT/mTOR pathway inhibitors on cisplatin resistance, migration and overall survival in cells expressing *PIK3CA*-E545K, to determine whether these phenotypes are an inherent property of the cell signaling pathways in these cells or an effect exclusive to GDC-0941. Such experiments may also shed light on pathways of PI3K-mediated cisplatin resistance and enhanced migration in cervical cancer cells expressing the *PIK3CA*-E545K mutation.

In summary, our studies suggest that targeting the PI3K pathway may represent a novel and promising strategy that may provide therapeutic benefit in cervical cancer patients whose tumours express *PIK3CA*-E545K, by restoring cisplatin sensitivity and possibly reducing a more migratory potential. However, it is important to note that to date, experiments have been carried out only on established transformed cells growing in culture. To further test this hypothesis, it will be important to validate our findings in freshly acquired patient cells as well as animal models of cervical cancer.

## MATERIALS AND METHODS

### Cell culture

A panel of cervical cancer cell lines including SiHa and CaSki was purchased from ATCC (Supplementary Table S1). SiHa (*PIK3CA*-wt) and CaSki (*PIK3CA*-wt/*PIK3CA*-E545K) cell lines were cultured in MEM and RPMI (Invitrogen), respectively, supplemented with 10% (v/v) fetal bovine serum (FBS, Hyclone III, Invitrogen) and penicillin (50 units/ml) and streptomycin (50 μg/ml). HeLa cells (*PIK3CA*-WT) were cultured in DMEM with 5% (v/v) serum and antibiotics as above. Stable cell lines derived from parental HeLa cell line (described below and in Supplementary Material) were cultured in DMEM supplemented with 5% fetal bovine serum, streptomycin and penicillin as above and other antibiotics as indicated below. All cell lines were cultured in a humidified incubator under an atmosphere of 5% CO_2_ at 37°C. SiHa, CaSki and HeLa cell lines were authenticated by STR analysis by ATCC January 2016.

### Confirmation of *PIK3CA* status in cell lines

Total DNA was extracted from cell lines using a DNeasy Blood and Tissue Kit (QIAGEN, catalogue No. 69506) and *PIK3CA* status was confirmed as described previously [27] (Supplementary Table S1).

### Transfection and generation of stable cell lines

To deplete *PIK3CA*, HeLa cells were transfected with pRS retroviral vector encoding one of four unique shRNA sequences to *PIK3CA* (OriGene catalogue number: TR310428). Twenty-four hours after transfection, cells were placed in DMEM containing 5% fetal bovine serum and 2.5 μg/ml puromycin (Sigma-Aldrich, MO, USA) and after two weeks, single cell populations were isolated and evaluated for PI3K protein expression by western blot. Based on low expression of p110α protein, a cell line expressing shRNA plasmid A, that we named A5, was selected for further study (Supplementary Figure S1).

For re-expression of shRNA-resistant *PIK3CA-WT* and *PIK3CA*-E545K, three mutations at the shRNA target site (Q981, C984, Y985) were generated in *PIK3CA* cDNA (bearing a C-terminal Myc and DDK tag) in the pCMV6 Entry vector (OriGene, Catalogue number RC213112) using a Quikchange Site Directed Mutagenesis kit (Stratagene). Mutation of E545K was carried out in a similar manner. A5 cells were then transfected with vectors for shRNA-resistant *PIK3CA*-WT, shRNA-resistant *PIK3CA*-E545K or empty vector as a control using Lipofectamine 2000 according to the manufacturers recommended conditions. The transfected cells were selected by growth in medium containing 2.5 μg/ml puromycin and 600 μg/ml G418 (Sigma-Aldrich, MO, USA). HeLa cells over-expressing *PIK3CA*-E545K (to create cells expressing both endogenous *PIK3CA*-WT and Myc, DDK-tagged *PIK3CA*-E545K) were generated in a similar manner except that selection was carried out using 400 μg/ml G418. After 14 days, single cell populations were isolated and characterized for relative expression of p110/*PIK3CA* by western blot. Cell lines A5-WT4, A5-E545K5 and HeLa-E545K (Supplementary Figure S1) were selected for further study. Mutations introduced into vectors were confirmed by sequencing at the University of Calgary DNA sequencing Centre. Primer sequences and detailed procedures are available upon request.

### Cell treatments

The PI3K/Akt inhibitor GDC-0941 was purchased from Selleck Chemicals, dissolved in 1 ml DMSO to a concentration of 10 mM and stored at −80°C. Cisplatin was purchased from Sigma, suspended in PBS (containing 154 mM NaCl) as described [29] and stored at −20°C. Where indicated, GDC-0941, cisplatin or appropriate controls, as described above, were added directly to the cell media at the concentrations indicated. Irradiation was carried out using a GammaCell1000 Elite ^137^Cs source (MDS Nordion) with a dose rate of approximately 2.9 Gy/min, as described previously [50].

### Immunoblots

Cells were lysed in an ice cold NETN lysis buffer [150 mM NaCl, 0.2 mM EDTA, 50 mM Tris-HCl (pH 7.5), and 1% (v/v) NP-40] containing protein phosphatase and protease inhibitors (1 μM microcystin-LR, 0.2 mM phenylmethylsulfonyl fluoride, 0.1 μg/mL pepstatin, 0.1 μg/mL aprotinin, and 0.1 μg/mL leupeptin), and lysed on ice by sonication (2 x 5 s bursts using a Fisher Scientific Sonic Dis-membrator Model 100). Total protein [50 μg; as determined by the Detergent-Compatible Protein Assay (Bio- Rad, CA, USA) using bovine serum albumin as standard] was resolved by SDS-PAGE and electrophoretically transferred onto nitrocellulose membranes. Membranes were blocked with 20% (w/v) skim milk powder in T-TBS buffer [20 mM Tris-HCl (pH 7.5), 500 mM NaCl, and 0.1% (v/v) Tween 20] for 1 hour and probed with antibodies to total proteins or phosphorylated proteins as indicated. Antibodies to p110 beta, Myc, PTEN, total RSK, RSK pSer 380 and total Akt were purchased from Cell Signaling. Antibodies to p85 and Ku80 were from Abcam, and the antibody to Akt pSer473 was from Santa Cruz. The antibody to p110α was kindly provided by Prof. Bart Vanhaesebroeck and Dr. Mariona Graupera (Centre for Cell Signaling, University of London, UK). Western blots were washed in TTBS and after incubation with the appropriate secondary antibody, and developed using ECL reagent (Perkin Elmer) and Fuji X-Ray film. All experiments were repeated at least 3 times. For quantitation, immunoblots were scanned at 300 dpi grayscale, quantitated using Image J software and normalized to Ku80 (loading control). The mean of three experiments with SEM is shown.

### Clonogenic survival assays

Cells were trypsinized, seeded in 4 mL medium and incubated overnight before irradiation or treatment with cisplatin or GDC-0941, as described below. After 14 days, cells were fixed then stained with crystal violet as described previously [50] and colonies (greater than 50 cells) were counted. Survival curves were plotted using Prism 6.0 Software (GraphPad). Where indicated, cells were irradiated with 1, 2, 4, or 6 Gy IR or incubated with increasing doses of cisplatin (0.1, 0.5, 1 or 2 μM) made up in PBS or an equal volume of PBS as vehicle control. GDC-0941 or an equal volume of DMSO (to give a final concentration of 0.01%) was added at 0.01, 0.1 and 0.5 μM. Unless otherwise indicated, cisplatin was removed after 24 hours. Each experiment was carried out in triplicate and results from three separate experiments are shown.

### Serum starvation

Stable cells expressing the *PIK3CA*-E545K mutation and/or wild-type *PIK3CA* were plated on 10 cm dishes in DMEM supplemented with 5% (v/v) fetal bovine serum and incubated for 24 hours. The media was then replaced with DMEM supplemented with 2% (v/v) serum for 48 hours. The concentration of serum in the media was then reduced to 0.2% (v/v) and cells were harvested and cell lysates were prepared after an additional 48 hours incubation.

### Wound healing assay

Cells were plated in 6 cm tissue culture dishes and grown to confluency. After 24hrs cells were either left untreated or treated with 1 μM GDC-0941 (formulated in DMSO) or an equivalent volume of DMSO (final concentration 0.01%) as control, treated with radiation (2 Gy) and cisplatin (formulated in PBS) at 1 μM or treated with 1 μM GDC-0941 (formulated in DMSO) radiation (2 Gy) and cisplatin (formulated in PBS) at 1 μM. 24hrs after treatment, scratches were made with a sterile 200 μL pipette tip and wound healing was observed 0, 6, 24, and 48 hours after initiation of the scratch. At different time points, images were generated using a Carl Zeiss Axiovert 200M microscope at 10 x magnification and imported into Photoshop Creative Suite, version 6.0. The rate of migration was measured by quantifying the total distance between the edges of the scratch as described previously [51]. Each experiment was repeated at least 3 times.

### Transwell migration assays

Transwell migration experiment was performed in HeLa, CaSki and HeLa-E545K cells using 24-well Transwell Boyden chamber system (8 μm-pore size, Corning, New York, USA). Cells were either left untreated or treated with 1 μM GDC-0941 (formulated in DMSO), or an equivalent volume of DMSO (final concentration 0.01%) as control, treated with radiation (2 Gy) and cisplatin (formulated in PBS) at 1 μM or treated with 1 μM GDC-0941 (formulated in DMSO) radiation (2 Gy) and cisplatin (formulated in PBS) at 1 μM. Cells were seeded in the top chamber to perform transwell migration assay. Media supplemented with 10% FBS was used as a chemoattractant in the lower chamber. The cells were incubated for 24hr at 37°C. Non-migrated cells were removed from the upper face of the transwell insert using a cotton swab. Cells that migrated through membranes were stained with 1% crystal violet, 95% ethanol and then counted. The results are representative of three independent experiments.

### Statistical analysis

Results are expressed as means ± SEM. For Figures 1–6, statistical significance was determined using multiple t-tests with the Holm-Sidak method to correct for multiple comparisons. For Figure 7, ordinary one-way ANOVA with no matching or pairing and Tukey's multiple comparisons test was used. Statistical analysis was performed using GraphPad Prism 6.0 Software. *p* values of <0.05 were considered statistically significant.

## SUPPLEMENTARY FIGURES and TABLE


