# Supplementary material for: Phosphatidyl inositol-3 kinase (PIK3CA) E545K mutation confers cisplatin resistance and a migratory phenotype in cervical cancer cells
Source: Oncotarget. 2016 Jul 30;7(50):82424–39. doi: 10.18632/oncotarget.10955 (PMC5347702; doi:10.18632/oncotarget.10955)
Supplement: Supplementary file 1 [file oncotarget-07-82424-s001.pdf]

Phosphatidyl inositol-3 kinase (*PIK3CA*) E545K mutation confers cisplatin resistance and a migratory phenotype in cervical cancer cells

SUPPLEMENTARY FIGURES AND TABLE

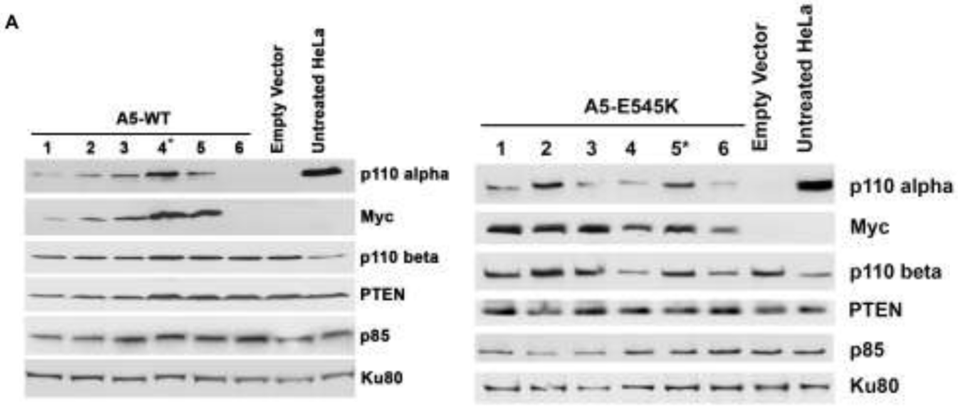

Supplementary Figure S1A: Generation of stable cell lines. Western blots of individual clones of A5 cells (HeLa cells depleted for *PIK3CA*, clone 5, A5), re-expressing either shRNA resistant *PIK3CA-WT* (left) or shRNA resistant *PIK3CA*-E545K (right). A5 cells were transfected with shRNA resistant *PIK3CA*-wt, *PIK3CA*-E545K or empty vector as control and stable cell lines were generated by selection on G418 and puromycin (double antibiotic resistance). Expression of *PIK3CA* was assessed by western blot and probing for the Myc tag on the expressed protein. Cell lines A5-WT4 and A5-E545K5 (indicated by *) were selected for further study.

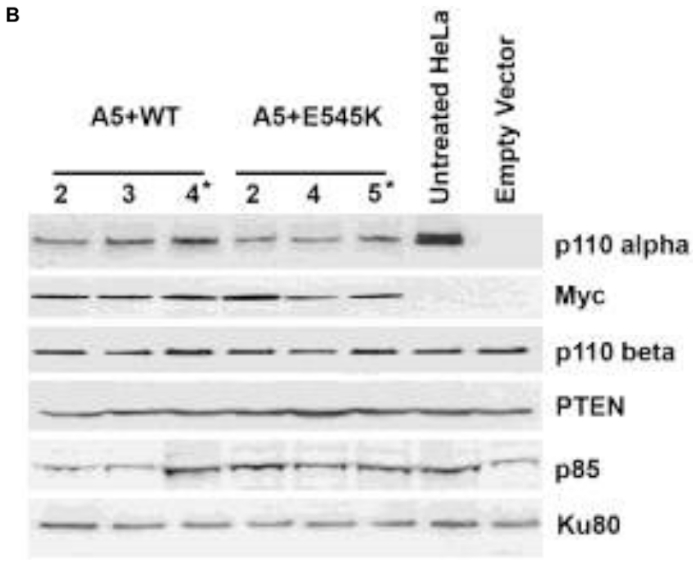

Supplementary Figure S1B: Generation of stable cell lines. Western blots comparing expression of shRNA resistant *PIK3CA*-wt and *PIK3CA*-E545K on the same gel. Comparison of the expression level of *PIK3CA* (p110-alpha) in the cell lines A5-WT and A5-E545K on the same gel in order to directly compare expression levels. Cell lines A5-WT4 and A5-E45K5 (indicated by *) were used in future experiments.

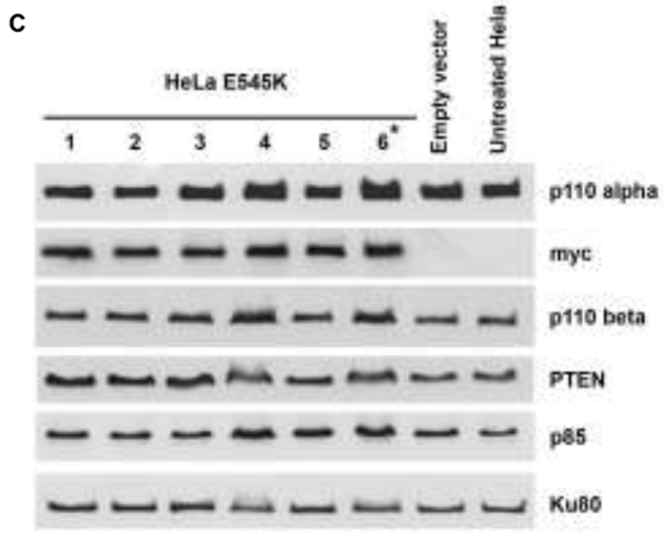

Supplementary Figure S1C: Generation of stable cell lines. Western blots of individual cell lines of HeLa cells (wt-*PIK3CA*) stably overexpressing *PIK3CA*-E545K. HeLa cells were transfected with *PIK3CA*-E545K and stable cell lines were generated by selection on G418 (400 µg/ml). Sequencing showed that all clones were positive for the E545K mutation. Cell extracts were analyzed by western blot with the antibodies indicated on the right. Further experiments were carried out with clone 6 (indicated by *).

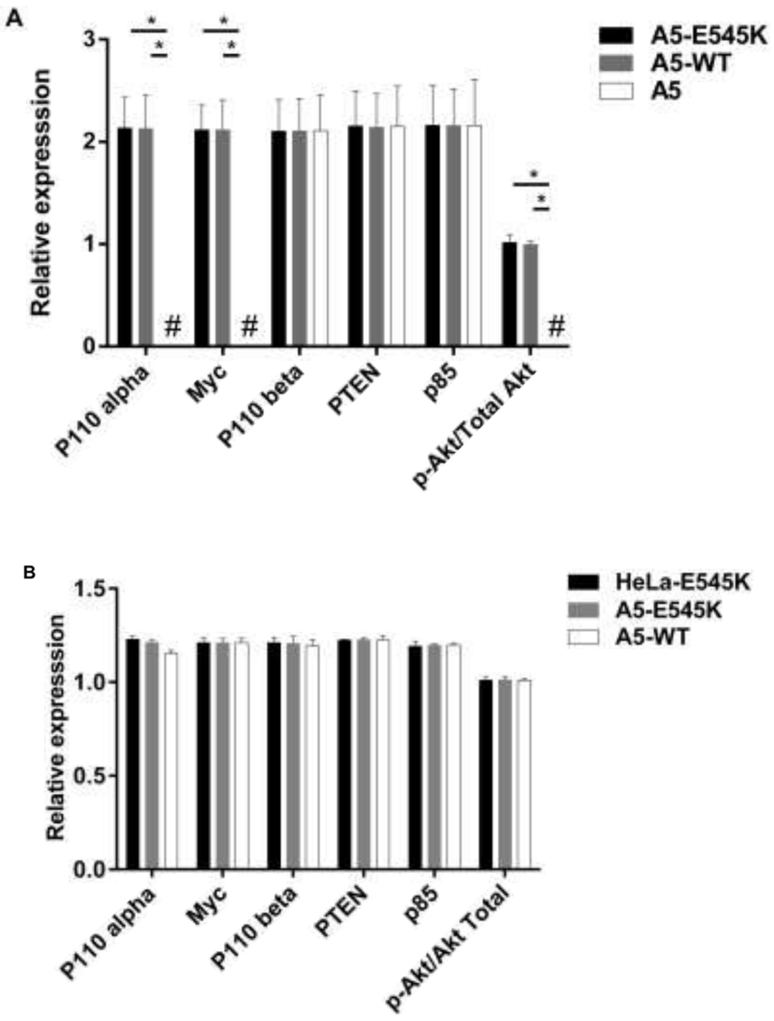

Supplementary Figure S2: Quantitation of western blots shown in Figures 2A & 3A. A. Quantitation of western blots in Figure 2A showing expression of p110α, p110β, PTEN, p85 and Akt/pS473-Akt in A5, A5-wt and A5-E545K cell lines. In each experiment, signals on western blot were normalized to Ku80 (loading control) and the mean from 3 separate experiments is shown. Black bars indicate A5-E545K cells, gray bars A5-WT cells and white bars A5 cells. The presence of the # symbol indicates where the white bars are below the limits of detection (e.g. p110α, Myc and pAkt)/total Akt). Error bars denote S.E.M. statistical significance was determined using multiple t-tests with the Holm-Sidak method to correct for multiple comparisons. p values for expression of p110α, Myc and phosphoAkt/Total Akt in A5-E545K cells (black bars) versus A5 (white bars) were 0.002, 0.0009 and 0.00018 respectively. p values for expression of p110α, Myc and phosphoAkt/Total Akt for A5-WT cells (gray bars) compared to A5 cells (white bars) were 0.0029, 0.0019 and <0.0001 respectively. P values < 0.05 were considered statistically significant and are indicated by the asterisks. **B.** Quantitation of western blots in Figure 3A showing expression of p110α, p110β, PTEN, p85 and Akt/pS473-Akt in HeLa-E545K (black bars), A5-E545K (gray bars) and A5-WT (white bars) cell lines. In each experiment, signals from western blot were normalized to Ku80 (loading control) and the mean from 3 separate experiments is shown. No statistically significant difference in protein expression was observed between the 3 cell lines.

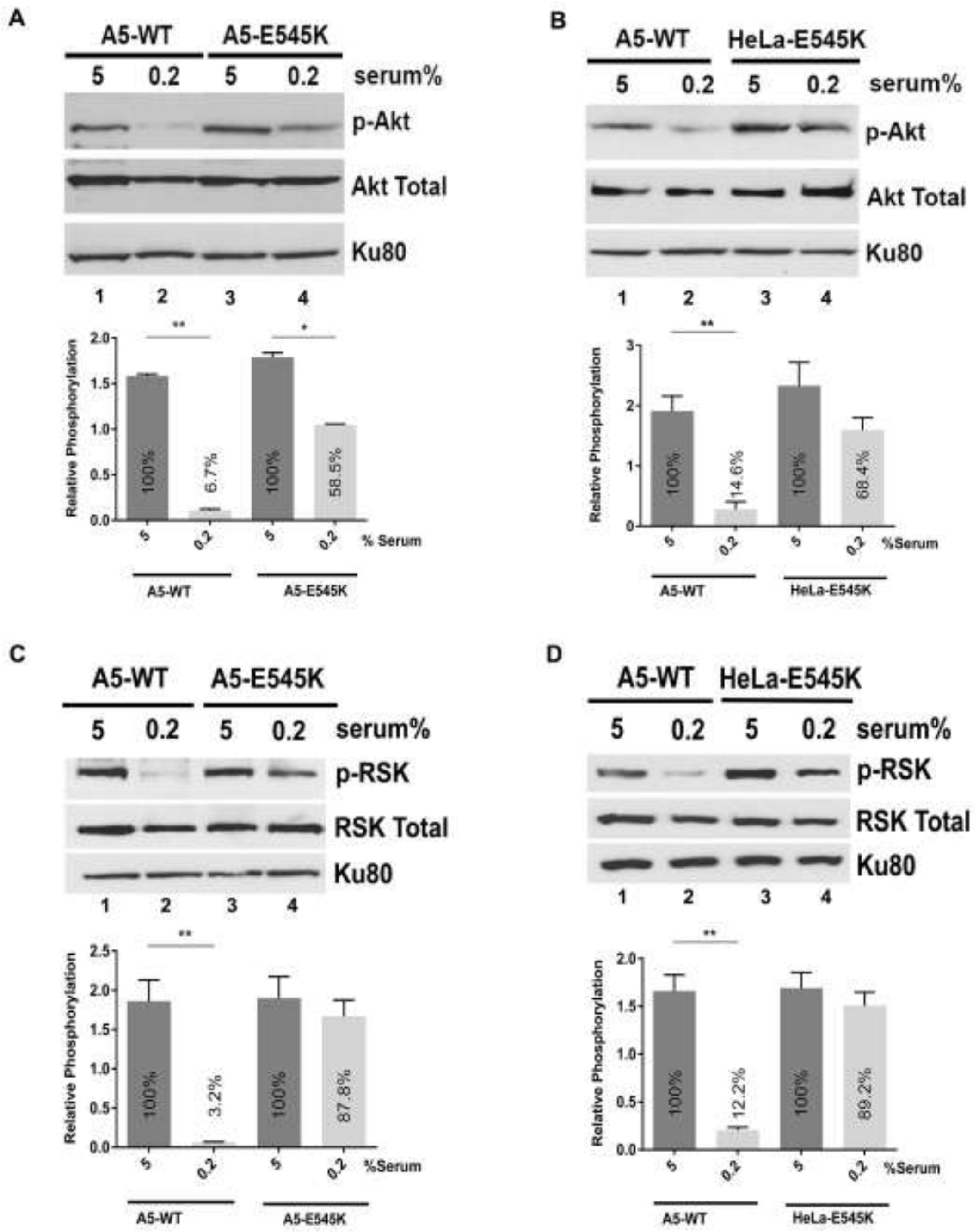

Supplementary Figure S3: Cervical cancer cells expressing *PIK3CA*-E545K retain residual Akt and RSK phosphorylation after serum starvation. A5-WT (HeLa cells with shRNA depletion of endogenous *PIK3CA* and stable expression of *PIK3CA*-WT), A5-E545K (HeLa cells with shRNA depletion of *PIK3CA* and stable expression of shRNA resistant *PIK3CA*-E545K, panels **A.** and **C.** and HeLa-E545K (HeLa cells stable expressing *PIK3CA*-E545K, panels **B.** and **D.** cells were grown in 5% serum then placed in media containing 2% serum for 48 hours, followed by media containing 0.2% serum for 48 hours. Extracts were made and assayed by western blot for either Akt- pS473 and total Akt (panels A and B) or RSK-pS380 and total RSK (panels C and D). In each experiment, phosphorylation of Akt or RSK was quantitated, normalized to Ku80 (loading control) and the mean from 3 separate experiments is shown below each representative experiment. Error bars denote S.E.M. The graphs show relative phosphorylation of Akt or RSK for each cell line at 5% and 0.2% serum. The percent phosphorylation at 0.2% serum relative to phosphorylation at 5% serum is indicated on the graphs. Statistical significance was determined using two-tailed un- paired t-tests. p values for changes in phosphorylation at 5% serum compared to 0.2% serum are for panel A: A5-E545K 0.0002; A5-WT, <0.0001; panel B, HeLa-E545K, 0.1732; A5-WT, 0.0045; panel C: A5-E545K, 0.5431 and A5-WT, 0.0026, and panel D, HeLa-E545K, 0.4485 and A5-WT, 0.0011. *p* values less than 0.05 are represented by * and less than 0.001 are represented by **.

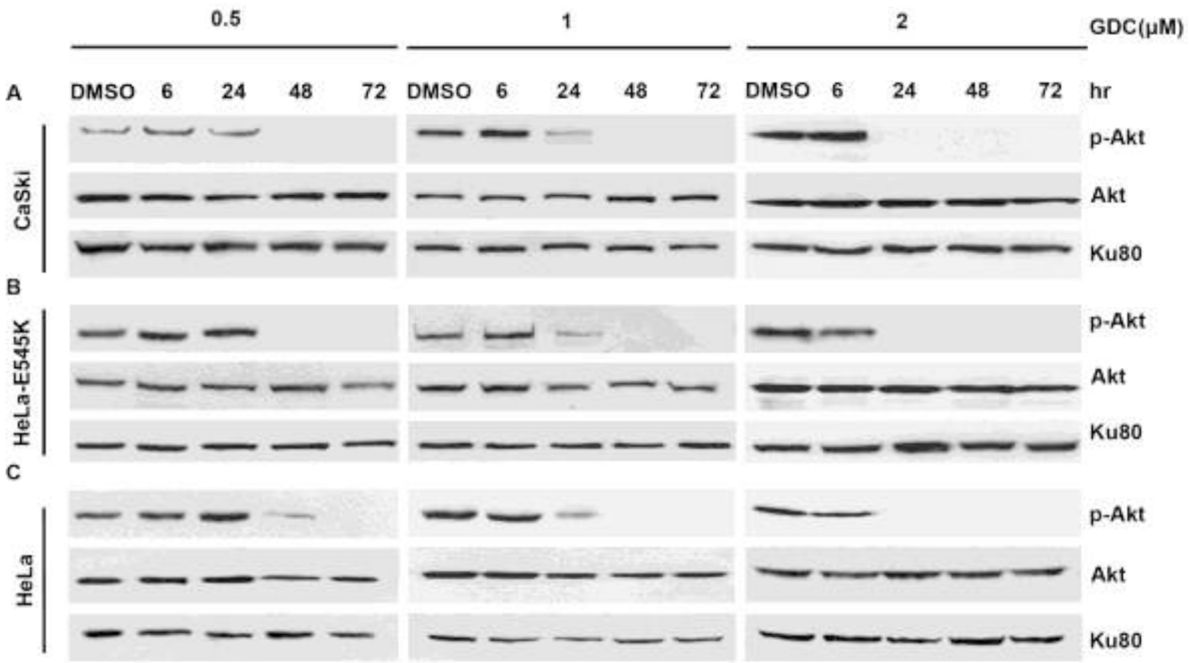

Supplementary Figure S4: Inhibition of Akt-S473 phosphorylation by GDC-0941. Asynchronously growing CaSki panel A. HeLa-E545K panel B. or HeLa cells panel C. were incubated with either DMSO alone (0.01%, lanes 1, 6 and 11), or GDC-0941 at 0.5 (lanes 2-5), 1 (lanes 7-10) or 2 µM (lanes 12-15) for 6, 24, 48 or 72 hours as indicated. Extracts were made and assayed by western blot as above. The results show that GDC-0941 inhibits Akt pS473 phosphorylation at all concentrations tested.

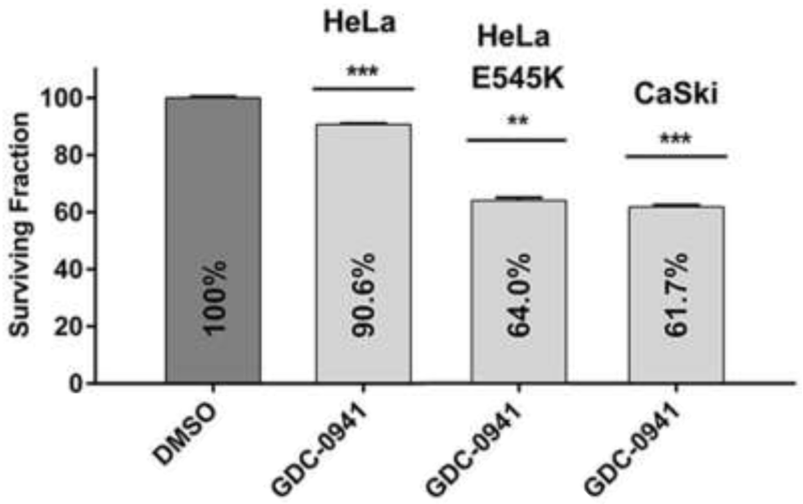

Supplementary Figure S5: Clonogenic cell survival assays in cervical cancer cell lines HeLa (*PIK3CA*-WT), CaSki (*PIK3CA*-WT plus *PIK3CA*-E545K) with GDC-0941 alone. Cervical cancer cell lines HeLa (*PIK3CA*-WT), HeLa-E545K (*PIK3CA*-WT plus *PIK3CA*-E545K) and CaSki (*PIK3CA*-WT plus *PIK3CA*-E545K) were seeded on 6 cm plates and 24 hours later incubated with either GDC-0941 (final concentration GDC 0.5 µM and DMSO 0.01%) or DMSO vehicle control (final concentration 0.01%). After 24 hours, media was replaced with fresh media and the plates were incubated at 37°C, under 5% CO_2. After 14 days, the plates were fixed, stained and colonies were counted as above. Results are expressed as mean ± SEM for each GDC-0941 treated cell line normalized to its own DMSO treated control. The % survival after GDC-0941 treatment for each cell line (relative to its own DMSO control) is shown in the graph. Statistical significance was determined using a two-tailed homoscedastic *t*-test. All results were highly significant, *p* values < 0.001 are indicated by ** and <0.0001 by ***.

A

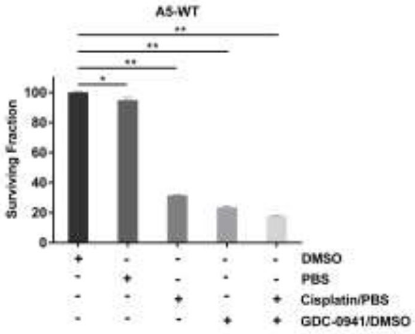

B

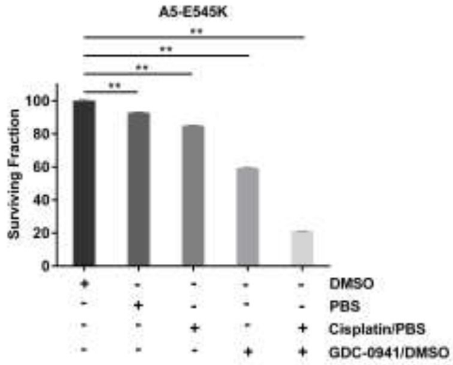

C

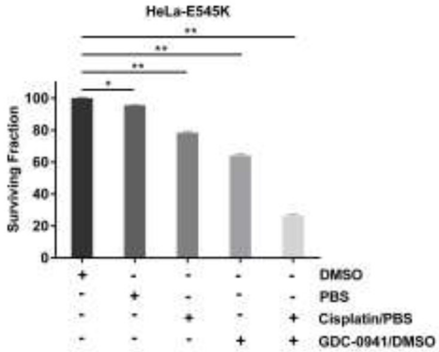

Supplementary Figure S6: GDC-0941 restores cisplatin sensitivity in *PIK3CA*-E545K expressing cells. HeLa-A5 cells transfected with shRNA resistant *PIK3CA*-WT (A5-WT), A5 cells transfected with shRNA-resistant *PIK3CA*-E545K (A5-E545K) and HeLa cells overexpressing *PIK3CA*-E545K (HeLa-E545K) were seeded on 6 cm plates and 24 hours later treated with either control PBS (154 mM NaCl) or cisplatin (formulated in PBS) at 1 µM and GDC-0941 at 0.5 µM respectively. After 24 hours, media was replaced with fresh media and plates were incubated at 37°C, under 5% CO_2. After 14 days, the plates were fixed, stained and colonies were counted as above. The results show the means of three separate experiments with standard deviation. Statistical significance was determined using ordinary one-way ANOVA with Dunnett's multiple comparison test. For each cell lines different treatment conditions were compared to the control group. In A5-WT, DMSO vs PBS, the p value was <0.05. For DMSO vs. Cisplatin/PBS and DMSO, vs. GDC-0941/DMSO, and vs. Cisplatin/PBS and GDC-0941/DMSO, the p value was <0.0001. In A5-E545K, DMSO vs PBS, vs. Cisplatin/PBS and DMSO, vs. GDC-0941/DMSO, and vs. Cisplatin/PBS and GDC-0941/DMSO, the p value was <0.0001. In HeLa-E545K, DMSO vs PBS, the p value was <0.01. For DMSO vs. Cisplatin/PBS and DMSO, vs. GDC-0941/DMSO, and vs. Cisplatin/PBS and GDC-0941/DMSO, the p value was <0.0001.

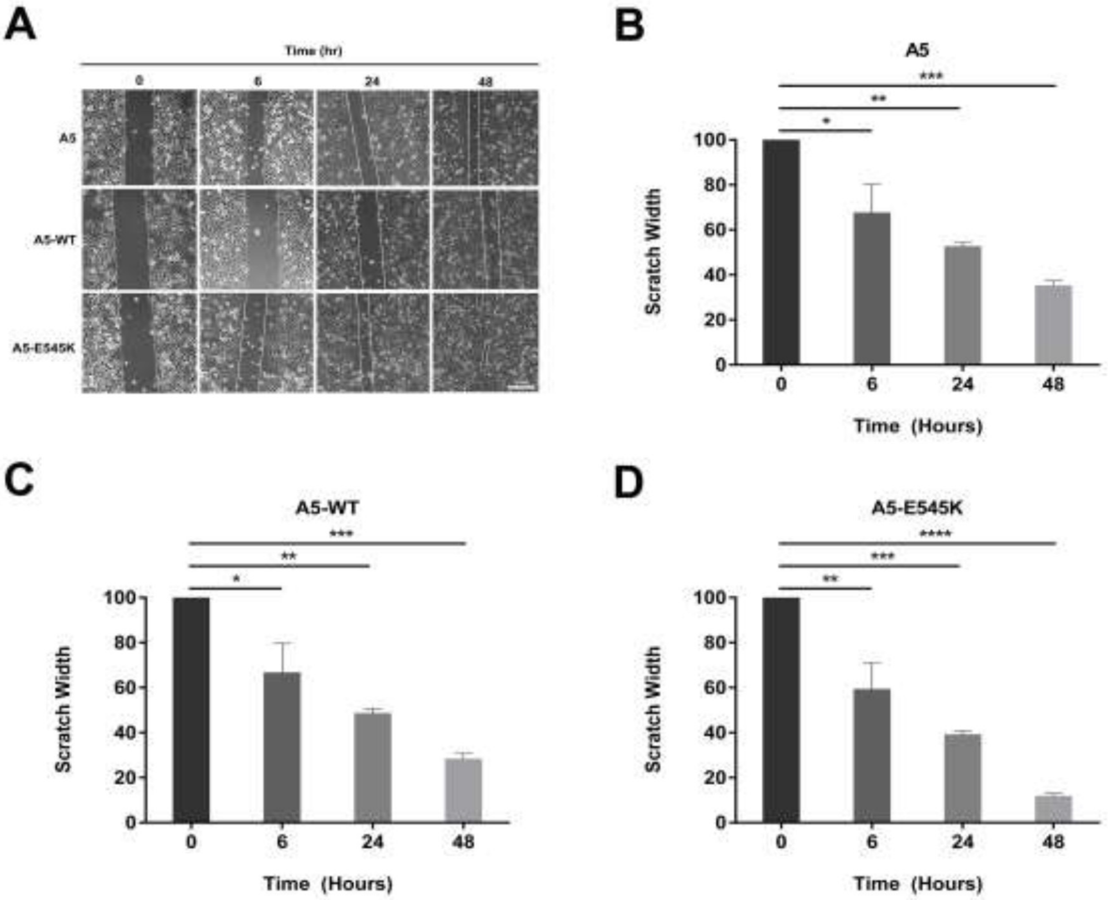

Supplementary Figure S7: Cells expressing *PIK3CA*-E545K have a more migratory phenotype. A. A5, A5-WT and A5-E545K cells were grown to confluency on 6 cm plates. Scratches were made and wound healing was observed 0, 6, 24, and 48 hours after initiation of the scratch. The rate of migration was measured by quantifying the total distance between the edges of the scratch at each time point. Scale bars = 100 µm. **B.** Average scratch widths, normalized to the width at 0 hours, from three separate experiments with standard deviation are shown using ordinary one way ANOVA with Dunnett's multiple comparison test. All the time points were compared to the 0hr time point. For A5 at 0hr vs 6hr is *, 0hr vs 24hr is ** and 0hr vs 48hr is *** respectively. *p* values of <0.05 are represented by *, <0.01 represents ** and <0.001 represents *** respectively. **C.** Average scratch widths, normalized to the width at 0 hours, from three separate experiments with standard deviation are shown using ordinary one way ANOVA with Dunnett's multiple comparision test. All the time points were compared to the 0hr time point. For A5-WT at 0hr vs 6hr is *, 0hr vs 24hr is ** and 0hr vs 48hr is *** respectively. *p* values of <0.05 are represented by *, <0.01 represents ** and <0.001 represents *** respectively. **D.** Average scratch widths, normalized to the width at 0 hours, from three separate experiments with standard deviation are shown using ordinary one way ANOVA with Dunnett's multiple comparision test. All the time points were compared to the 0hr time point. For A5-E545K at 0hr vs 6hr is **, 0hr vs 24hr is *** and 0hr vs 48hr is **** respectively. *p* values of <0.01 represents **, <0.001 represents *** and <0.0001 represents **** respectively.

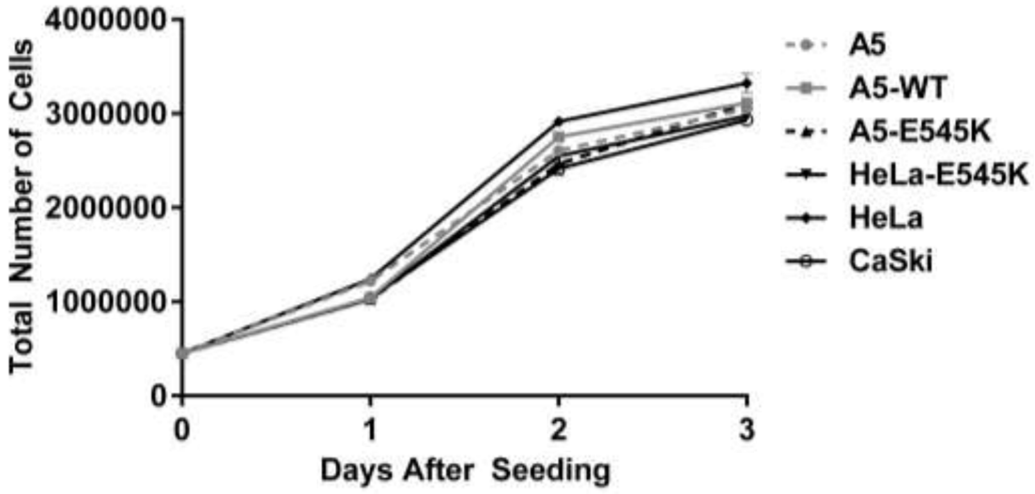

Supplementary Figure S8: Growth curves for HeLa, CaSki and A5, A5-WT, A5-E545K and HeLa-E545K cell lines. Asynchronously growing cells were harvested 1, 2 or 3 days after plating and the number of cells counted using a hemocytometer after trypan blue staining. Growing conditions for each cell line are described in Material and Methods.

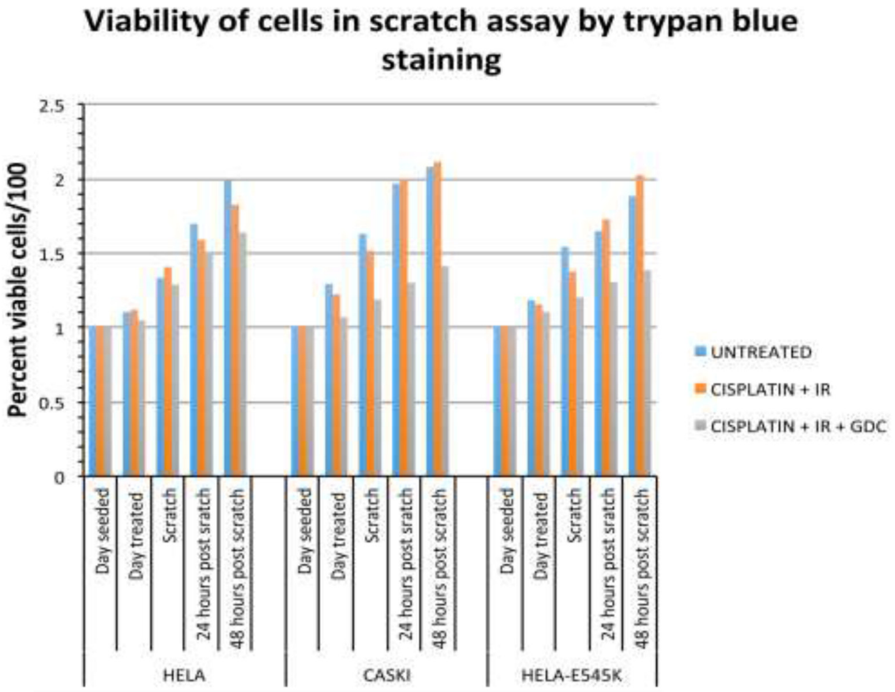

Supplementary Figure S9: An approximately equal number (1.7 x 10^6) of HeLa, Hela-E545K and CaSki cells were seeded on 6 cm plates. After 24 hours, cells were either left untreated or treated with 1 µM GDC-0941 (formulated in DMSO) or an equivalent volume of DMSO (final concentration 0.01%) as control, treated with radiation (2 Gy) and cisplatin (formulated in PBS) at 1 µM or treated with 1 µM GDC-0941 (formulated in DMSO) radiation (2 Gy) and cisplatin (formulated in PBS) at 1 µM. After twenty four hours media was removed and replaced with fresh media and scratches were made with a sterile 200 µL pipette tip. Cells were harvested by 24 and 48 hours later as indicated in the figure. In each case, an aliquot of cells was mixed with trypan blue (0.4%) and the percentage of viable cells was determined using automated cell counter (Bio-Rad TC10). At the 48 hour time point, the viability of HeLa cells treated with cisplatin plus IR was 91.2% compared to the mock treated controls. For HeLa cells treated with cisplatin/IR and GDC, the % viability was 82%. For CaSki cells the % viability was 102% (minus GDC) and 68% plus GDC and for HeLa-E545K, the viability was 108% minus GDC and 74% plus GDC. The results suggest that GDC-0941 reduces the viability of cells bearing the *PIK3CA*-E545K mutation by approximately 30% compared to cells with *PIK3CA*-wt. Whether this is due to reduced proliferation or enhanced cell death remains to be determined.

Supplementary Table S1: Characteristics of the cervical cell lines used in this study

A. Cell lines purchased from ATCC:

**Cell line (ATCC nomenclature)**	**HPV status**	***PIK3CA* status**	**Origin**
HeLa (CCL2)	type18	wt	Cervix adenocarcinoma
SiHa (HTB-35)	type16	wt	Cervix squamous cell carcinoma
CaSki (CRL-1550)	many copies of type 16 and possibly 18	E545K heterozygous	Cervix squamous cell carcinoma Metastasized (small intestine)

B. Stable cell lines generated and used in this study:

A5	HeLa with shRNA depletion of *PIK3CA*
A5-WT	A5 with stable expression of shRNA-resistant *PIK3CA*-WT
A5-E545K	A5 with stable expression of shRNA-resistant *PIK3CA*-E545K
HeLa-E545K	HeLa with stable expression of *PIK3CA*-E545K

HeLa, SiHa and CaSki cells were obtained from ATCC. Information on HPV status, p53 protein expression, *PIK3CA* status and cell lines origin is from ATCC. A5, A5-WT, A5-E545K and HeLa-E545K were generated as described in Materials and Methods and below. *PIK3CA* status was confirmed by DNA sequencing where indicated.
